# Association of Social Support With Oral Health in Older Adults: A Systematic Review and Meta‐Analysis

**DOI:** 10.1111/ger.70072

**Published:** 2026-05-15

**Authors:** Sungkrit Pojmonpiti, Saif S. Syed, Murali Srinivasan, Georgios Tsakos, Frauke Müller, Barbara Janssens

**Affiliations:** ^1^ ELOHA (Equal Lifelong Oral Health for All) Research Group, Department of Oral Health Sciences, Faculty of Medicine and Health Sciences University of Ghent Ghent Belgium; ^2^ Department of Neurology Ghent University Hospital Ghent Belgium; ^3^ Dental Department, Chulabhorn Hospital Chulabhorn Royal Academy Bangkok Thailand; ^4^ Department of Epidemiology and Public Health University College London London UK; ^5^ Institute of Dentistry Queen Mary University of London London UK; ^6^ Clinic of General, Special Care, and Geriatric Dentistry, Center for Dental Medicine University of Zurich Zurich Switzerland; ^7^ Center of Excellence in Genomics and Precision Dentistry, International Program in Geriatric Dentistry and Special Care, Faculty of Dentistry Chulalongkorn University Bangkok Thailand; ^8^ Division of Gerodontology and Removable Prosthodontics, University Clinics of Dental Medicine University of Geneva Geneva Switzerland; ^9^ Department of Oral Health Ghent University Hospital Ghent Belgium

**Keywords:** geriatric dentistry, meta‐analysis, oral health, social support, systematic review

## Abstract

**Background:**

Social support is an important determinant of health in later life, yet no study has synthesised the relevant evidence. This systematic review and meta‐analysis aimed to synthesise evidence on the association between social support (functional, structural, and perceived dimensions) and oral health among older adults aged 75 years and over.

**Methods:**

The review process, using a systematic approach and random‐effects meta‐analysis, was conducted in accordance with PRISMA guidelines. Searches were carried out in Medline, Embase, and CENTRAL up to November 2025. Eligible studies examined associations between social support and oral health, including dentition status, oral conditions, oral function, oral health behaviours, and oral health–related quality of life. Risk of bias was assessed using the Newcastle–Ottawa Scale.

**Results:**

Forty studies were included. Meta‐analyses showed that low functional support—defined as having fewer people to rely on—was associated with edentulism (OR 1.21, 95% CI: 1.15, 1.27) and non‐regular dental service use (OR 1.20, 95% CI: 1.17, 1.23). Low structural support, in terms of living conditions, was associated with edentulism and lacking functional dentition (OR 1.34, 95% CI: 1.02, 1.75; and OR 1.41, 95% CI: 1.20, 1.67, respectively). The evidence on the associations between low social support and other oral health measures was sparse and heterogeneous.

**Conclusion:**

Poor social support was associated with poor dentition status and oral health behaviours among older adults aged 75 years and over. These findings underscore the importance of incorporating psychosocial factors into oral health assessment and care planning for this population.

**Trial Registration:**

PROSPERO; registration number: CRD420251231319

## 1 Introduction

Oral health is an essential component of healthy ageing, influencing nutrition and quality of life [1]. In older adults, oral conditions often coexist with other age‐related health conditions, such as cognitive decline and multimorbidity, which further complicate their oral health needs and limit their ability to perform daily self‐care [2]. Moreover, there are stark inequalities in the oral health of older adults [3, 4]. These arise from complex interactions among a wide range of interrelated factors, including behaviours, psychosocial factors and biology [5, 6, 7]. Psychosocial factors refer to social conditions (e.g., work conditions) and interpersonal relationships (e.g., social networks and support) that shape individuals' attitudes or behaviours [8]. They are recognised as important determinants of health [9], and some of them, such as chronic stress, have been associated with poor oral health [7, 10], while others, such as social relationships and social support, have been shown to promote healthy behaviours and support overall health and well‐being [11, 12].

Social support is defined as the network of resources—either received or available—from family, friends, and community relationships that provide emotional, practical, and informational assistance [13]. It operates through interconnected physiological, psychosocial, and behavioural pathways, influencing stress regulation, coping capacity, and care‐seeking behaviours [12, 13, 14, 15, 16, 17, 18, 19, 20, 21]. Social support presents a multidimensional construct that overlaps across disciplines, reflecting its conceptual breadth and methodological complexity [13, 17, 22]. Common taxonomies distinguish between structural and functional support, as well as between received and perceived support [13, 17, 22]. Functional support reflects the assistance received and the quality or depth of relationships [13, 17, 22], whereas structural support refers to the size of the social network, the frequency of interactions, and living arrangements [13, 17, 22]. In addition, received support overlaps with both functional and structural support, as it involves assistance provided when needed, whereas perceived support reflects the expectation that help will be available during times of need and can be conceptualised through constructs such as loneliness [13, 17, 22, 23]. Social support is closely related to other psychosocial constructs, more specifically to social isolation at the individual level, while social relationships and social capital span both individual and community levels [17, 22, 24].

In older adults, social support plays a vital role in oral health, particularly when they become dependent. It is associated with healthier behaviours such as regular toothbrushing and dental attendance. Being married or cohabiting, a measure of structural support, is frequently linked with healthier behaviours that contribute to better oral health [18, 25, 26, 27]. Similarly, social capital is built and utilised over time, but may decline due to life transitions such as retirement and reduced mobility, leading to a higher risk of social isolation and loneliness, which in turn are associated with poorer oral health behaviours and oral health‐related quality of life (OHRQoL) [28, 29, 30]. Older adults aged 75 years and older are a population group that experiences increased frailty, functional decline, and social vulnerability. While age thresholds such as 60 or 65 years have been used to define older populations, geriatric syndromes and complex care needs become increasingly prevalent in the oldest‐old [31]. In this age group, adaptive capacity may be reduced, leading to greater reliance on external social support, potentially amplifying the relevance and influence of social support on oral health [32, 33]. Therefore, this study focused on the particularly vulnerable group of older adults aged ≥ 75 years.

To date, no study has synthesised the evidence on the association between social support and a wide range of oral health measures for older adults aged 75 years or older. Previous systematic reviews have included broad age ranges, selected only specific related psychosocial constructs (social isolation, loneliness, or social relationships), but did not comprehensively focus on social support, and covered only specific oral health measures, such as dentition status or oral disease [24, 28, 29]. In response to this gap in the literature, this systematic review and meta‐analysis aimed to synthesise evidence on the association between social support—including functional, structural, and perceived dimensions—and oral health among older adults aged 75 years and over.

## 2 Methods

This systematic review and meta‐analysis was conducted and reported in accordance with the Preferred Reporting Items for Systematic Reviews and Meta‐Analyses (PRISMA) guidelines [34]. The protocol was registered in the International Systematic Review Registry (PROSPERO; registration number: CRD420251231319).

### 2.1 Search Strategy

A comprehensive search was conducted in Medline (PubMed), Embase and the Cochrane Central Register of Controlled Trials (CENTRAL) to identify relevant studies without language restrictions, covering publications up to November 2025. Medical Subject Headings (MeSH), keywords, and other free‐text terms related to the PECO framework (Table 1) were used to address the research question: ‘What is the association between social support and oral health among older adults?’ The same keywords were applied across all databases, with syntax adjusted as needed according to the specific requirements of each platform (Appendix I).

**TABLE 1:** PECO framework.

Component	Description
Population (P)	Older adults aged ≥ 75 years (including studies with a mean age of approximately 75 years)
Exposure (E)	Lack of social support, including limited or no assistance from family, friends, caregivers, community organisations or healthcare professionals
Comparison (C)	Older adults with adequate or strong social support
Outcomes (O)	Oral health includes clinical indicators, functional measures, and behavioural factors (e.g., dentition status, oral diseases and functions, and self‐reported oral health)

### 2.2 Eligibility Criteria

Studies were eligible for inclusion if they assessed the association between social support and oral health among older adults aged 75 years or older. This also included studies that enrolled participants younger than 75 years if they reported extractable data specifically for those aged 75 years and above, or if the reported mean age of participants was 74.5 years or older. Eligible studies included observational or interventional designs that provided quantitative data on oral health measures. To ensure a sufficient sample size, studies were required to include at least 10 participants per group. Studies were excluded if they lacked measurable or descriptive information on oral health, did not assess social support, or were case reports or case series.

### 2.3 Study Selection and Data Extraction

All references retrieved from the electronic databases were imported into the web‐based application Rayyan (www.rayyan.ai) for duplicate removal, screening, and selection. Two reviewers (S.P. and S.S.S.) independently screened titles, abstracts, and full texts based on the predefined eligibility criteria. For studies that appeared to meet the eligibility criteria but were published in languages other than English, translations were generated using DeepL.com, an AI‐driven online translation tool, to support accurate data extraction and interpretation. Inter‐investigator reliability was evaluated using Cohen's unweighted kappa (*κ*) to maintain consistency in the screening process [35]. Any disagreements were resolved through discussion with the research team.

Data extraction was performed independently by two reviewers for each included study, collecting information on authors, year of publication, country or countries, study objectives, design, type of extracted data, type of population (community‐dwelling, institutionalised, or mixed), age, sample size, social support exposures, and oral health measures. The oral health measures were classified into five domains: dentition status; oral conditions (including dental caries, periodontal disease, and dry mouth); oral function; oral health behaviours; and OHRQoL. These data were then compiled into an evidence table. To ensure inclusion of all relevant data on the associations between social support and oral health, the extraction process covered both the key results and the baseline population characteristics reported in each study.

### 2.4 Risk of Bias Assessment

The risk of bias in the included studies was independently assessed by two reviewers (S.P. and S.S.S.). The assessment was conducted based on the type of data used in this review: the Newcastle–Ottawa Scale (NOS) was used for cohort data [36], and an adapted version of the NOS for cross‐sectional data [37].

### 2.5 Data Synthesis

In addition to the adjusted and unadjusted analyses reported, data from the descriptive baseline Tables of the included studies that could be used to examine associations between social support and oral health were analysed, using unadjusted odds ratios (ORs) for dichotomous variables and mean differences (MDs) for continuous variables. Where possible, these data were pooled in a meta‐analysis using random‐effects modelling to account for between‐study variability, with 95% confidence intervals (CIs) and assessments of heterogeneity. These results were further stratified by population to provide greater granularity. Studies were excluded from the meta‐analysis if only adjusted estimates were available (due to heterogeneity in covariates and modelling approaches), if insufficient information was provided to derive corresponding unadjusted estimates, or if they used convenience sampling. These studies were instead summarised narratively in the text.

The potential for publication bias was evaluated across the included studies using a funnel plot, which was generated only when at least five studies were available. The funnel plot also informed sensitivity analyses by identifying studies that had the greatest influence on the results in cases of moderate to high heterogeneity. For studies using the same participant sample, only data from the most recent publication were included in the meta‐analysis. All analyses were conducted in RStudio (version 4.5.0) using the meta package.

## 3 Results

**FIGURE 1:**
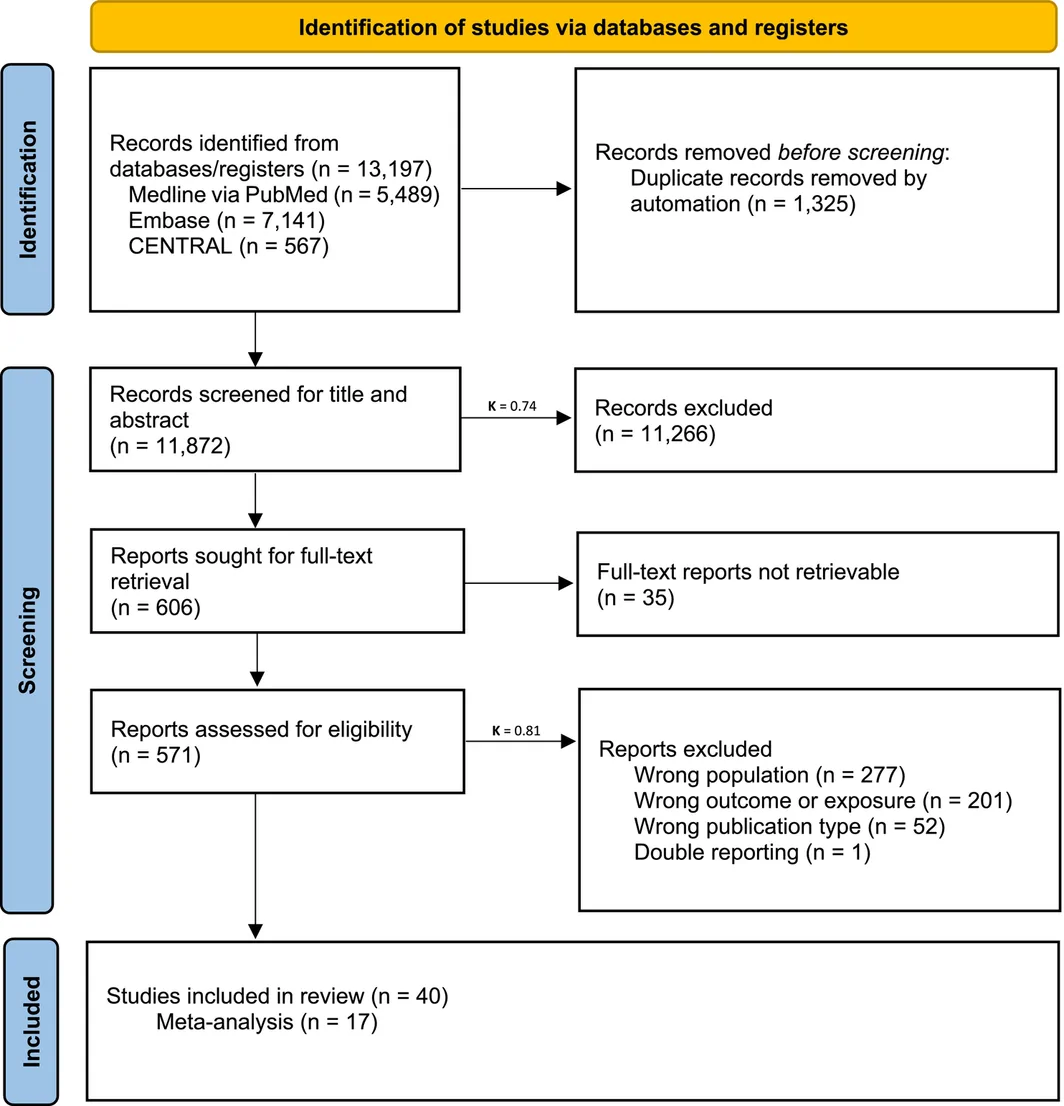
PRISMA 2020 flow diagram of study selection. [Colour figure can be viewed at wileyonlinelibrary.com]

Figure 1 illustrates the study selection process, beginning with 13,197 records identified across three databases and resulting in 11,872 records after removal of duplicates. The screening process showed good inter‐investigator agreement, with kappa coefficients of 0.74 for title and abstract screening and 0.81 for full‐text assessment. Details of the excluded studies and the reasons for exclusion are provided in Appendix II.

### 3.1 Study Characteristics

A total of 40 studies were included in this review [38, 39, 40, 41, 42, 43, 44, 45, 46, 47, 48, 49, 50, 51, 52, 53, 54, 55, 56, 57, 58, 59, 60, 61, 62, 63, 64, 65, 66, 67, 68, 69, 70, 71, 72, 73, 74, 75, 76, 77]. Although several studies were longitudinal in design, the majority of extracted data were cross‐sectional, with three studies providing cohort data [39, 46, 64]. One included study was in Japanese [74]. The study populations predominantly comprised community‐dwelling older adults, with a smaller number of studies conducted among institutionalised populations (*n* = 5) [45, 53, 54, 62, 66] or mixed populations comprising both community‐dwelling and institutionalised participants (*n* = 11) [42, 43, 57, 60, 64, 65, 70, 73, 75, 76, 77]. Social support was conceptualised and measured in three main ways in the included studies: functional support, structural support, and perceived support. In addition, marital status and living arrangements—considered part of structural support—were extracted from baseline characteristics in most studies (*n* = 29) [39, 40, 41, 43, 44, 45, 47, 48, 49, 50, 51, 52, 53, 54, 55, 56, 57, 59, 61, 65, 66, 67, 70, 71, 72, 73, 75, 76, 77]. Most studies assessed social support at the individual level, while two studies evaluated community‐level social support, specifically neighbourhood social capital [38] and the perceived neighbourhood quality index [46]. An overview of all included studies is presented in Table 2.

**TABLE 2:** Included studies with the association of social support and oral health in older adults.

Authors, year	Country	Study design	Type of data	Population	Age mean (SD)	Sample size^a^	Social support factors	Oral health domains				
								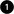	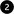	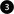	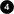	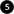
Aida et al., 2011 [38]	Japan	Longitudinal and cross‐sectional	Cross‐sectional	Community‐dwelling	74.9 (6.6)	21,736	[Functional and structural social support] Neighbourhood social capital: civic network, sports and hobby network, volunteer network, friendship network, social support Individual social support and network: civic network, sports and hobby network, volunteer network, friendship network, social support	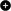				
Avlund et al., 2003 [39]	Sweden	Cohort	Cohort	Community‐dwelling	> or equal 80	129	[Structural social support] Frequency of contacts and function of social relations: weekly contacts, having no confidence or having lost confidence, satisfaction with frequency of contacts, satisfaction with social contacts [Living condition]: Living alone		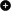			
Brothwell et al., 2008 [40]	Canada	Cross‐sectional	Cross‐sectional	Community‐dwelling	76.2 (7.1)	1751	[Structural social support] Number of helper/companions/people to count [Living condition]: Marital status				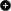	
Castrejón‐Pérez et al., 2017 [41]	Mexico	Longitudinal and cross‐sectional	Cross‐sectional	Community‐dwelling	79.2 (7.1)	654	[Living condition]: Marital status					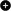
Chan et al., 2025 [42]	China	Cross‐sectional	Cross‐sectional	Community‐dwelling and institutionalised	82.6 (11.2)	7796	[Functional and structural social support] Social capital: social participation, social support (structural social support, emotional social support, instrumental social support), and trust in community	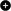				
Dai et al., 2023 [43]	China	Cohort and cross‐sectional	Cross‐sectional	Community‐dwelling and institutionalised	85.4 (10.5)	5403	[Living condition]: Living alone	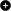				
Edman et al., 2016 [44]	Sweden	Cross‐sectional	Cross‐sectional	Community‐dwelling	equal 75, equal 85	879	[Living condition]: Living alone		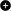			
Ferreira et al., 2008 [45]	Brazil	Cross‐sectional	Cross‐sectional	Institutionalised	79.0 (9.1)	335	[Living condition]: Marital status	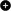				
Ganbavale et al., 2024 [46]	Britain	Longitudinal and cross‐sectional	Cohort and cross‐sectional	Community‐dwelling	78.7	2137	[Perceived social support] Perceived neighbourhood quality index including local area service (social and leisure facilities, health service, transport, etc.), safety environment (volume of traffic, noise, crime, air quality, etc.) and green spaces	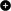	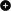			
Hatta et al., 2018 [47]	Japan	Cohort	Cross‐sectional	Community‐dwelling	between 79 ‐ 81	463	[Structural social support] Frequency of going out, frequency of interacting [Living condition]: Living alone	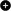				
Heegard et al., 2011 [48]	Denmark	Longitudinal and cross‐sectional	Cross‐sectional	Community‐dwelling	75.5	783	[Structural social support] Diversity in social relations (children, grand/great‐grandchildren, relatives, confidants, acquaintances and neighbours) with whom the participant had personal contact at least once a week Social participation including paying visits to others, receiving visits at home and participation in social activities outside the home [Living condition]: Living alone	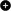				
Holm‐Pedersen et al., 2006 [49]	Sweden	Longitudinal and cross‐sectional	Cross‐sectional	Community‐dwelling	> or equal 80	101	[Living condition]: Marital status		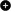			
Hsu et al., 2014 [50]	Taiwan	Cross‐sectional	Cross‐sectional	Community‐dwelling^b^	76.0 (0.4)	332	[Living condition]: Living alone					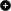
Hunt et al., 1985 [51]	US	Cross‐sectional	Cross‐sectional	Community‐dwelling	> or equal 75	3634	[Living condition]: Marital status	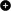				
Inomata et al., 2017 [52]	Japan	Longitudinal and cross‐sectional	Cross‐sectional	Community‐dwelling	between 79 ‐ 81	760	[Living condition]: Living alone	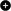				
Islas‐Granillo et al., 2016 [53]	Mexico	Cross‐sectional	Cross‐sectional	Institutionalised	79.1 (9.8)	139	[Living condition]: Marital status	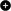				
Islas‐Granillo et al., 2014 [54]	Mexico	Cross‐sectional	Cross‐sectional	Institutionalised	79.1 (9.8)	139	[Living condition]: Marital status		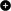			
Jensen et al., 2008 [55]	US	Cross‐sectional	Cross‐sectional	Community dwelling	79.1 (7.5)	641	[Living condition]: Living alone					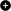
Kim et al., 2018 [56]	Korea	Cross‐sectional	Cross‐sectional	Community‐dwelling	82.7	241	[Functional and structural social support] Social capital in network dimensions (Lubben Network Scale): general network (relatives/friends you see or hear), friend network (relatives/friends you can talk to about private matters), supportive network (relatives/friends you can ask for help) [Living condition]: Living alone	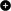		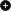		
Kim et al., 2009 [57]	Korea	Cross‐sectional	Cross‐sectional	Community‐dwelling and institutionalised	75.4 (7.7)	409	[Living condition]: Marital status					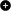
Komiyama et al., 2016 [58]	Japan	Cohort	Cross‐sectional	Community‐dwelling	75.2 (4.4)	834	[Functional social support] Social support (someone they can rely on for emotional support, advice, help with daily tasks, transportation to medical care, and caregiving when ill.)	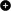			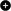	
Liang et al., 2020 [59]	Taiwan	Cross‐sectional	Cross‐sectional	Community‐dwelling^b^	79.0	166	[Living condition]: Marital status			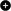		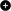
Lundgren et al., 1995 [60]	Sweden	Cross‐sectional	Cross‐sectional	Community‐dwelling and institutionalised	equal 88	354	[Perceived social support] Feelings of loneliness		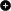	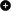	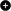	
Musacchio et al., 2007 [61]	Italy	Longitudinal and cross‐sectional	Cross‐sectional	Community‐dwelling	76.8 (8.7)	3054	[Living condition]: Living alone	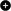				
Niesten et al., 2017 [62]	Netherlands	Cross‐sectional	Cross‐sectional	Institutionalised	85.4 (7.1)	126	[Functional social support] ENRICHD social support index Social support to go to dentist				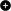	
Osterberg et al., 1995 [63]	Sweden, Denmark, Finland	Cross‐sectional	Cross‐sectional	Community‐dwelling	equal 75	945	[Structural social support] Social support index based on frequency of contacts with children, grandchildren, neighbours and friends Social activity index based on frequency of travel, membership of associations	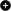				
Qi et al., 2023 [64]	China	Cohort and cross‐sectional	Cohort	Community‐dwelling and institutionalised	80.4 (9.7)	4268	[Functional and structural social support] Social isolation including living alone, were not/no longer married, lacked social support and did not participate in social activities. [Perceived social support] Loneliness by asking do you feel lonely	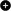				
Rapp et al., 2017 [65]	France	Cross‐sectional	Cross‐sectional	Community‐dwelling and institutionalised	82.5 (6.3)	1314	[Living condition]: Marital status		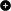			
Rocha‐Ortiz et al., 2023 [66]	Mexico	Cross‐sectional	Cross‐sectional	Institutionalised^b^	81.3 (9.0)	256	[Living condition]: Marital status	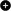				
Syrjälä et al., 2010 [67]	Finland	Cross‐sectional	Cross‐sectional	Community‐dwelling	> or equal 75	523	[Living condition]: Marital status	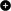				
Takahashi et al., 2023 [68]	Japan	Longitudinal and cross‐sectional	Cross‐sectional	Community‐dwelling	> or equal 75	5490	[Functional and structural social support] Social isolation using Lubben Social Network Scale [Perceived social support] Loneliness Scale version 3 of University of California, Los Angeles (UCLA)	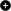		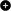	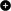	
Takeda et al., 2025 [69]	Japan	Cross‐sectional	Cross‐sectional	Community‐dwelling	> or equal 75	4196	[Structural social support] Social participation including “Going out” and “meeting with family or friends”	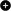	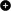	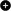	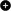	
Thorstensson et al., 2010 [70]	Sweden	Longitudinal and cross‐sectional	Cross‐sectional	Community‐dwelling and institutionalised	> or equal 80	357	[Living condition]: Marital status	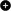				
Tsakos et al., 2009 [71]	UK	Cross‐ sectional	Cross‐sectional	Community‐dwelling	74.7	1043	[Living condition]: Living alone					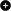
Wright et al., 2018 [72]	Australia	Longitudinal and cross‐sectional	Cross‐sectional	Community‐dwelling	> or equal 78	524	[Living condition]: Marital status	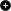	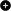			
Wu et al., 2018 [73]	China	Cross‐sectional	Cross‐sectional	Community‐dwelling and institutionalised	75.3 (6.7)	195	[Living condition]: Living alone					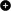
Yanagisawa et al., 2023 [74]	Japan	Cross‐sectional	Cross‐sectional	Community‐dwelling	75.1 (6.9)	739	[Perceived social support] Social support appraisals instrument measuring perceived emotional support	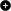				
Yang et al., 2022 [75]	China	Cohort and cross‐sectional	Cross‐sectional	Community‐dwelling and institutionalised	> or equal 80	5513	[Living condition]: Marital status	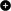				
Zini et al., 2008 [76]	Israel	Cross‐sectional	Cross‐sectional	Community‐dwelling and institutionalised^b^	75.7 (8.1)	344	[Living condition]: Marital status					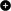
Zini et al., 2011 [77]	Israel	Case control and cross‐sectional	Cross‐sectional	Community‐dwelling and institutionalised^b^	75.7 (8.1)	344	[Living condition]: Marital status				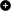	

Abbreviations: 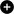: Oral health domains reported in the study, 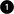: Dentition status,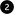: Oral conditions: dental caries, periodontal disease, dry mouth,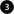: Oral function,
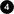
Oral health behaviours,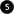:Oral health‐related quality of life.

^a^ Sample size reported based on the extracted data in this review.

^b^ Employed convenience samples.

### 3.2 Risk of Bias and Quality Assessment

The overall risk of bias among the cross‐sectional and cohort data ranged from low to moderate. Five studies were considered to have low population representativeness due to employing convenience sampling [50, 59, 66, 76, 77]. Detailed assessments are provided in Appendix III.

### 3.3 Synthesis of Results

Findings from the included studies and all meta‐analyses examining the association between social support and oral health are summarised in Table 3. Eight meta‐analyses were conducted. Of these, two (with edentulism and lacking functional dentition) had sufficient data for funnel plot evaluation and demonstrated an acceptable level of publication bias (Appendix IV: S1 and S2).

**TABLE 3:** Summary of findings on the association between social support and oral health in older adults.

Domains	Exposures	Study: *n*	Strength of association
Dentition status	Functional and/or structural social support	Ten studies [38]: 21,736 [42]: 7796 [47]: 463 [48]: 783 [56]: 241 [58]: 834 [63]: 945 [64]: 4,268 [68]: 5490 [69]: 4196	*Meta‐analysis (based on unadjusted data)* Edentulism versus low functional social support [56, 58]: OR 1.21, 95% CI 1.15 to 1.27 (Figure 3a) Number of teeth < 20 versus low functional social support [38, 58, 68]: OR 1.02, 95% CI 0.97 to 1.08 (Figure 4a); Low structural social support (social participation) [38, 48]: OR 1.35, 95% CI 0.02 to 112.2 (Figure 4b) *Unpooled analyses (adjusted) (n = 6)* Remaining teeth > 20 versus neighbourhood social capital (reference lowest): Highest civic network OR 0.89, 95% CI 0.89 to 1.10; Highest sports and hobby network OR 1.04, 95% CI 0.94 to 1.16; Highest volunteer network OR 0.92, 95% CI 0.82 to 1.03; Highest friendship network OR 1.17, 95% CI 1.04 to 1.30; Highest social support OR 1.01, 95% CI 0.92 to 1.10; Medium civic network OR 0.96, 95% CI 0.87 to 1.06; Medium sports and hobby network OR 1.02, 95% CI 0.93 to 1.12; Medium volunteer network OR 0.96, 95% CI 0.87 to 1.06; Medium friendship network OR 1.10, 95% CI 1.00 to 1.21; Medium social support OR 1.01, 95% CI 0.93 to 1.10 [38] Remaining teeth > 20 versus individual social support and network (reference lowest): Highest civic network OR 1.07, 95% CI 0.97 to 1.19; Highest sports and hobby network OR 1.12, 95% CI 1.02 to 1.22; Highest volunteer network OR 1.09, 95% CI 0.97 to 1.23; Highest friendship network OR 1.14, 95% CI 0.99 to 1.30; Highest social support OR 1.03, 95% CI 0.94 to 1.13; Medium civic network OR 1.01, 95% CI 0.91 to 1.12; Medium sports and hobby network OR 0.99, 95% CI 0.87 to 1.12; Medium volunteer network OR 0.98, 95% CI 0.88 to 1.10; Medium friendship network OR 1.14, 95% CI 1.04 to 1.25; Medium social support OR 0.93, 95% CI 0.82 to 1.05 [38] Tooth loss versus social participation *B* = −0.07, SE = 0.01, *p* ≤ 0.01 [42] Edentulism versus network social capital (reference 5 persons or more): General network less than 1 person PR 1.31, 95% CI 0.83 to 2.08; Friend network less than 1 person PR 0.79, 95% CI 0.52 to 1.22; Supportive network less than 1 person PR 0.81, 95% CI 0.53 to 1.25; General network 2–4 persons PR 0.83, 95% CI 0.54 to 1.26; Friend network 2–4 persons PR 0.74, 95% CI 0.50 to 1.09; Supportive network 2–4 persons PR 0.76, 95% CI 0.51 to 1.14 [56] Edentulism versus social support NS; Edentulism versus social activity *B* −0.09, SE = 0.04, *p* ≤ 0.01 [63] [7 years cohort data] Number of remaining teeth versus social isolation *B* −0.06, 95% CI −0.13 to 0.00, *p* ≤ 0.05 [64] Tooth loss versus socially isolated PR 0.97, 95% CI 0.93 to 1.01 [68] *Unpooled analyses (unadjusted) (n = 7)* Remaining teeth > 20 versus neighbourhood social capital (reference lowest): Highest civic network OR 1.03, 95% CI 0.93 to 1.14; Highest sports and hobby network OR 1.37, 95% CI 1.24 to 1.51; Highest volunteer network OR 1.10, 95% CI 0.99 to 1.22; Highest friendship network OR 1.41, 95% CI 1.28 to 1.56; Highest social support OR 1.00, 95% CI 1.00 to 1.00; Medium civic network OR 1.00, 95% CI 1.00 to 1.00; Medium sports and hobby network OR 1.22, 95% CI 1.10 to 1.35; Medium volunteer network OR 1.07, 95% CI 0.97 to 1.18; Medium friendship network OR 1.19, 95% CI 1.08 to 1.31; Medium social support OR 1.04, 95% CI 0.93 to 1.17 [38] Remaining teeth > 20 versus individual social support and network (reference lowest): Highest civic network OR 1.76, 95% CI 1.63 to 1.90; Highest sports and hobby network OR 1.93, 95% CI 1.80 to 2.07; Highest volunteer network OR 2.01, 95% CI 1.84 to 2.20; Medium civic network OR 1.49, 95% CI 1.37 to 1.62; Medium sports and hobby network OR 1.61, 95% CI 1.45 to 1.79; Medium volunteer network OR 1.61, 95% CI 1.47 to 1.76 [38] Tooth loss versus social capital (total scores) *r* = −0.069, *p* ≤ 0.01: Social participation *r* = −0.245, *p* ≤ 0.01; Social support *r* = −0.077, *p* ≤ 0.01 [42] No posterior occlusal support versus social network: Low frequency of going out (0–2 times in a week) OR 1.66, 95% CI 1.08 to 2.54; Low frequency of interacting (0–1 time in a month) OR 0.83, 95% CI 0.53 to 1.28 [47] Number of teeth < 20 versus low social participation OR 0.94, 95% CI 0.71 to 1.25 [48] Edentulism versus network social capital (reference 5 persons or more): General network less than 1 person PR 2.13, 95% CI 1.45 to 3.14; Friend network less than 1 person PR 1.09, 95% CI 0.70 to 1.71; General network 2–4 persons PR 1.04, 95% CI 0.69 to 1.55; Friend network 2–4 persons PR 0.89, 95% CI 0.59 to 1.35 [56] Number of remaining teeth versus social isolation *B* −0.05, 95% CI −0.11 to 0.00, *p* ≤ 0.05; Number of remaining teeth versus loneliness *B* −0.01, 95% CI −0.05 to −0.04, NS [64] Number of remaining teeth versus low social participation: MD −3.34, 95% CI −4.61 to −2.07 (going out), MD −2.07, 95% CI −3.52 to −0.62 (meeting family or friends) [69]
Dentition status	Perceived social support	Four studies [46]: 1722 [64]: 4268 [68]: 5490 [74]: 739	*Unpooled analyses (adjusted) (n = 3)* Edentulism versus social support based on perceived neighbourhood quality index (reference first Q, least problems): OR 1.06, 95% CI 0.51 to 2.19 (second Q); OR 1.68, 95% CI 0.80 to 3.51 (third Q); OR 1.98, 95% CI 1.00 to 3.94 (fourth Q); OR 1.28, 95% CI 0.58 to 2.80 (fifth Q, most problems); *p* for trend 0.12 [46] [8 years cohort data] Tooth loss versus social support based on perceived neighbourhood quality index (reference first Q, least problems): OR 1.36, 95% CI 0.83 to 2.20 (second Q); OR 1.42, 95% CI 0.83 to 2.43 (third Q); OR 1.71, 95% CI 1.01 to 2.90 (fourth Q); OR 1.89, 95% CI 1.00 to 3.57 (fifth Q, most problems); *p* for trend 0.02 [46] [7 years cohort data] Number of remaining teeth versus loneliness *B* 0.15, 95% CI −0.01 to 0.30, NS [64] Tooth loss versus lonely PR 1.06, 95% CI 1.01 to 1.11 [68] *Unpooled analyses (unadjusted) (n = 2)* Number of teeth < 20 versus lonely OR 1.44, 95% CI 1.17 to 1.78 [68] Oral health (subjective sense of health, number of remaining teeth, and oral function) versus perceived social support *r* = 0.121, *p* ≤ 0.01 [74]
Dentition status	Living conditions	Fourteen studies [43]: 5403 [45]: 335 [47]: 463 [48]: 766 [51]: 3634 [52]: 760 [53]: 139 [56]: 241 [61]: 3054 [66]: 256^a^ [67]: 523 [70]: 357 [72]: 524 [75]: 5513	*Meta‐analysis (based on unadjusted data)* Mean number of missing teeth versus living alone or unmarried [53, 72]: MD 1.58, 95% CI −1.29 to 4.45 (Figure 2) Edentulism versus living alone or unmarried [43, 45, 51, 56, 61, 67]: OR 1.34, 95% CI 1.02 to 1.75 (Figure 3b, Appendix IV: S3) Number of teeth < 20 versus living alone or unmarried [43, 48, 61, 67, 75]: OR 1.41, 95% CI 1.20 to 1.67 (Figure 4c, Appendix IV: S5) *Unpooled analyses (adjusted) (n = 3)* Edentulism versus living alone: NS (men); OR 1.47, 95% CI 1.15 to 1.88 (women) [61] Number of teeth (between 0 teeth and ≥ 20 teeth) versus married OR 3.6, 95% CI 1.3 to 9.7, *p* = 0.013; Number of teeth (between 1–10 teeth and ≥ 20 teeth) versus never married OR 12.2, 95% CI 1.6 to 94.2, *p* = 0.016 [70] Number of teeth < 20 versus unmarried: OR 1.06, 95% CI 0.75 to 1.50 (males); OR 1.21, 95% CI 0.82 to 1.78 (females) [75] *Unpooled analyses (unadjusted) (n = 3)* No posterior occlusal support versus living alone OR 0.72, 95% CI 0.45 to 1.16 [47] Low number of teeth (0–9 teeth) versus living alone OR 0.68, 95% CI 0.46 to 0.99 [52] Mean number of missing teeth versus living alone MD 2.69, 95% CI −1.26 to 6.64 [66]
Oral conditions: dental caries, periodontal disease, dry mouth	Functional and/or structural social support	Two studies [39]: 129 [69]: 4196	*Unpooled analyses (adjusted) (n = 1)* [7 years cohort data] Having root caries versus sustained dissatisfied with frequency of social contact OR 2.9, 95% CI 1.2 to 7.2 [39] *Unpooled analyses (unadjusted) (n = 2)* [7 years cohort data] Having coronal caries versus social network: Sustained no weekly contacts OR 0.6, 95% CI 0.3 to 1.3; Sustained having no confidant or having lost confidant OR 0.9, 95% CI 0.4 to 1.8; Sustained low or decrease in satisfaction with frequency of contacts OR 0.7, 95% CI 0.3 to 1.5; Sustained low or decrease in satisfaction with social contacts OR 0.6, 95% CI 0.3 to 1.2 [39] [7 years cohort data] Having root caries versus social network: Sustained no weekly contacts OR 1.1, 95% CI 0.5 to 2.3; Sustained having no confidant or having lost confidant OR 0.9, 95% CI 0.5 to 1.9; Sustained low or decrease in satisfaction with frequency of contacts OR 2.7, 95% CI 1.2 to 6.1; Sustained low or decrease in satisfaction with social contacts OR 1.4, 95% CI 0.7 to 3.2 [39] Poor oral hygiene status versus low social participation: OR 2.07, 95% CI 1.42 to 3.00 (going out), OR 2.24, 95% CI 1.49 to 3.38 (meeting family or friends); Dry mouth versus low social participation: OR 1.63, 95% CI 1.15 to 2.32 (going out), OR 1.06, 95% CI 0.67 to 1.68 (meeting family or friends) [69]
Oral conditions: dental caries, periodontal disease, dry mouth	Perceived social support	Two studies [46]: 1722 [60]: 354	*Unpooled analyses (adjusted) (n = 2)* 20% sites affected by periodontal pockets > 3.5 mm versus social support based on perceived neighbourhood quality index (reference first Q, least problems): OR 1.39, 95% CI 0.80 to 2.41 (second Q); OR 1.09, 95% CI 0.58 to 2.07 (third Q); OR 1.09, 95% CI 0.59 to 2.02 (fourth Q); OR 1.90, 95% CI 1.00 to 3.66 (fifth Q, most problems); *p* for trend 0.22 [46] Xerostomia versus social support based on perceived neighbourhood quality index (reference first Q, least problems): OR 0.94, 95% CI 0.61 to 1.45 (second Q); OR 1.54, 95% CI 0.93 to 2.43 (third Q); OR 1.50, 95% CI 0.93 to 2.42 (fourth Q); OR 2.31, 95% CI 1.31 to 4.09 (fifth Q, most problems); *p* for trend < 0.001 [46] [8 years cohort data] Xerostomia versus social support based on perceived neighbourhood quality index (reference first Q, least problems): OR 1.02, 95% CI 0.65 to 1.59 (second Q); OR 1.46, 95% CI 0.87 to 2.44 (third Q); OR 1.64, 95% CI 0.98 to 2.73 (fourth Q); OR 2.03, 95% CI 1.12 to 3.68 (fifth Q, most problems); p for trend = 0.002 [46] Xerostomia versus feelings of loneliness *B* = 0.10, NS [60]
Oral conditions: dental caries, periodontal disease, dry mouth	Living conditions	Six studies [39]: 129 [44]: 879 [49]: 101 [54]: 139 [65]: 1314 [72]: 524	*Unpooled analyses (adjusted) (n = 2)* [7 years cohort data] Having coronal caries versus sustained living alone or having become alone OR 2.4, 95% CI 1.0 to 5.7 [39] Serious periodontitis versus married OR 1.33, 95% CI 0.44 to 4.04 [49] *Unpooled analyses (unadjusted) (n = 6)* [7 years cohort data] Having coronal caries versus sustained living alone or having become alone OR 1.7, 95% CI 0.8 to 3.6; Having root caries versus sustained living alone or having become alone OR 0.7, 95% CI 0.4 to 1.6 [39] Having caries lesion versus single living OR 1.02, 95% CI 0.77 to 1.34 [44] Serious periodontitis versus married OR 2.82, 95% CI 1.16–6.90 [49] Saliva flow (amount) versus marital status *p* = 0.747 [54] Oral health using OHAT versus marital status *p* = 0.019; Oral health using OHAT versus Living situation *p* = 0.010 [65] Number of decayed teeth versus Married MD −0.13, 95% CI −0.42 to 0.16; Number of decayed root surfaces versus married MD −0.3, 95% CI −1.28 to 0.65; Number of decayed and/or filled root surface versus married MD −0.78, 95% CI −5.93 to 4.37; Percentage of tooth sites with pocket depth ≥ 3 mm versus Married MD − 1.67, 95% CI − 3.08 to −0.27; Clinical attachment loss ≥ 5 mm versus married MD − 3.40, 95% CI − 5.10 to −1.70; Gingival index ≥ 2 versus Married MD 0.28, 95% CI −1.41 to 1.97 [72]
Oral function	Functional and/or structural social support	Three studies [56]: 241 [68]: 5490 [69]: 4196	*Unpooled analyses (adjusted) (n = 2)* Poor chewing ability versus network social capital (reference 5 persons or more): General network less than 1 person PR 1.88, 95% CI 1.16 to 3.06; Friend network less than 1 person PR 1.62, 95% CI 0.93 to 2.83; Supportive network less than 1 person PR 1.54, 95% CI 0.89 to 2.65; General network 2–4 persons PR 1.18, 95% CI 0.78 to 1.79; Friend network 2–4 persons PR 1.36, 95% CI 0.81 to 2.27; Supportive network 2–4 persons PR 1.29, 95% CI 0.77 to 2.15 [56] Difficulty in chewing versus social isolation PR 1.63, 95% CI 1.10 to 2.41 [68] *Unpooled analyses (unadjusted) (n = 3)* Poor chewing ability versus network social capital (reference 5 persons or more): General network less than 1 person PR 2.27, 95% CI 1.38 to 3.73; Friend network less than 1 person PR 1.77, 95% CI 0.99 to 3.19; Supportive network less than 1 person PR 1.59, 95% CI 0.89 to 2.83; General network 2–4 persons PR 1.52, 95% CI 0.97 to 2.40; Friend network 2–4 persons PR 1.70, 95% CI 0.99 to 2.93; Supportive network 2–4 persons PR 1.52, 95% CI 0.89 to 2.60 [56] Cannot chew versus social isolated OR 2.30, 95% CI 1.62 to 3.26 [68] Masticatory function versus low social participation: MD − 5.50, 95% CI − 7.18 to −3.82 (going out), MD − 3.83, 95% CI − 5.78 to −1.88 (meeting family or friends); Swallowing function versus low social participation: MD 0.67, 95% CI −0.14 to 1.48 (going out), MD 0.33, 95% CI −0.61 to 1.27 (meeting family or friends); Poor tongue mobility versus low social participation: OR 2.32, 95% CI 0.52 to 10.43 (going out), OR 0.65, 95% CI 0.04 to 10.95 (meeting family or friends); Poor articulation Pa, Ta, Ka, Ta versus low social participation: NS (going out), NS (meeting family or friends) [69]
Oral function	Perceived social support	Two studies [60]: 354 [68]: 5490	*Unpooled analyses (adjusted) (n = 2)* Chewing problem versus feelings of loneliness *B* = 0.08, NS [60] Difficulty in chewing versus loneliness PR 2.94, 95% CI 1.96 to 4.43 [68] *Unpooled analyses (unadjusted) (n = 1)* Cannot chew versus lonely OR 4.18, 95% CI 2.88 to 6.08 [68]
Oral function	Living conditions	Two studies [56]: 241 [59]: 166^a^	Unpooled analyses (unadjusted) (*n* = 2) Poor chewing ability versus living alone OR 1.56, 95% CI 0.90 to 2.71 [56] Poor chewing ability versus unmarried/divorce/widow OR 1.79, 95% CI 0.87 to 3.71 [59]
Oral health behaviours	Functional and/or structural social support	Five studies [40]: 1751 [58]: 785 [62]: 126 [68]: 5490 [69]: 4196	*Meta‐analysis (based on unadjusted data)* No regular use of dental services versus low functional social support [58, 62]: OR 1.20, 95% CI 1.17 to 1.23 (Appendix IV: S6) *Unpooled analyses (adjusted) (n = 2)* Infrequent toothbrushing versus social isolation PR 1.13, 95% CI 0.95 to 1.35 [68] Brushing frequency versus ENRICHD social support index OR 0.97, 95% CI 0.91 to 1.04 [62] *Unpooled analyses (unadjusted) (n = 3)* Dental visitation versus number of helper/companions/people to count on NS (dentate, edentulous) [40] Not brushing teeth every day versus social isolated OR 1.51, 95% CI 1.26 to 1.83 [68] Not brushing teeth every day versus low social participation: OR 2.79, 95% CI 1.23 to 6.34 (going out), OR 2.60, 95% CI 1.01 to 6.69 (meeting family or friends) [69]
Oral health behaviours	Perceived social support	Two studies [60]: 354 [68]: 5490	*Unpooled analyses (adjusted) (n = 2)* Regular dental care versus loneliness OR 0.66, 95% CI 0.46 to 0.93 [60] Infrequent toothbrushing versus loneliness PR 1.50, 95% CI 1.23 to 1.83 [68] *Unpooled analyses (unadjusted) (n = 1)* Not brushing teeth every versus lonely OR 2.09 95% CI 1.67 to 2.61 [68]
Oral health behaviours	Living conditions	Two studies [40]: 1751 [77]: 344^a^	*Unpooled analyses (adjusted) (n = 2)* Dental visitation versus married NS (dentate, edentulous) [40] Dental care attendance versus married OR 0.37, 95% CI 0.18 to 0.74 [77] *Unpooled analyses (unadjusted) (n = 2)* Dental visitation versus married: NS (dentate); *p* ≤ 0.05 (edentulous); Dental visitation versus living alone NS (dentate, edentulous) [40] Dental care attendance versus married OR 0.34, 95% CI 0.16 to 0.72 [77]
OHRQoL	Living conditions	Eight studies [41]: 654 [50]: 332^a^ [55]: 641 [57]: 409 [59]: 166^a^ [71]: 1043 [73]: 195 [76]: 344^a^	*Meta‐analysis (based on unadjusted data)* Poor OHRQoL versus living alone or unmarried [41, 57, 71]: SMD 0.00, 95% CI −0.20 to 0.20 (Appendix IV: S7 and S8) *Unpooled analyses (adjusted) (n = 5)* Poor OHRQoL using GOHAI versus living alone *B* 2.33, 95% CI 0.38 to 4.27, *p* = 0.019 [50] Poor OHRQoL using OHIP‐14 versus married OR 2.00, 95% CI 0.99 to 4.06 [57] Better OHRQoL using GOHAI versus living alone NS [71] Better OHRQoL using GOHAI versus living with family *B* 0.69, 95% CI −2.83 to 4.21, *p* = 0.699 [73] Poor OHRQoL using OHIP‐14 versus family status OR 1.28, 95% CI 0.66 to 2.47, *p* = 0.46 [76] *Unpooled analyses (unadjusted) (n = 4)* Better OHRQoL using GOHAI versus cohabiting SMD 0.25, 95% CI −0.03 to 0.54 [50] Poor OHRQoL using OHIP‐14 versus living alone *r* = 0.034, *p* = 0.40 [55] Better OHRQoL using GOHAI versus unmarried/divorce/widow OR 0.63, 95% CI 0.33 to 1.20 [59] Better OHRQoL using OHIP‐14 versus cohabiting SMD 0.34, 95% CI 0.11 to 0.56 [76]

*Note:* Underline = Statistically significant.

Abbreviations: *B*, *B* coefficient; CI, confidence interval; GOHAI, Geriatric Oral Health Assessment Index; MD, mean difference; NS, non‐significant; OHAT, Oral Health Assessment Tool; OHIP‐14, Oral Health Impact Profile‐14; OHRQoL, Oral Health–Related Quality of Life; OR, odds ratio; PR, prevalence ratio; Q, quintile; *r*, Pearson correlation coefficient; SE, standard error; SMD, standardised mean difference.

^a^ Employed convenience sample.

#### 3.3.1 Dentition Status

A total of 23 studies assessed the relationship between social support and dentition status [38, 42, 43, 45, 46, 47, 48, 51, 52, 53, 56, 58, 61, 63, 64, 66, 67, 68, 69, 70, 72, 74, 75]. Six meta‐analyses were conducted.

**FIGURE 2:**
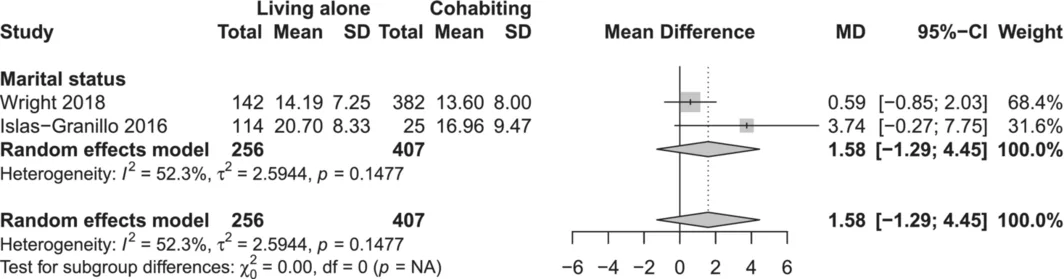
Forest plot of mean differences in the number of missing teeth by living conditions.

One meta‐analysis with moderate heterogeneity (*I* ^2^ = 52.3%) among the included studies of community‐dwelling [72] and institutionalised [53] populations showed a non‐significant difference in the mean number of missing teeth (MD 1.58, 95% CI: −1.29, 4.45) between those being single and those being married (Figure 2).

**FIGURE 3:**
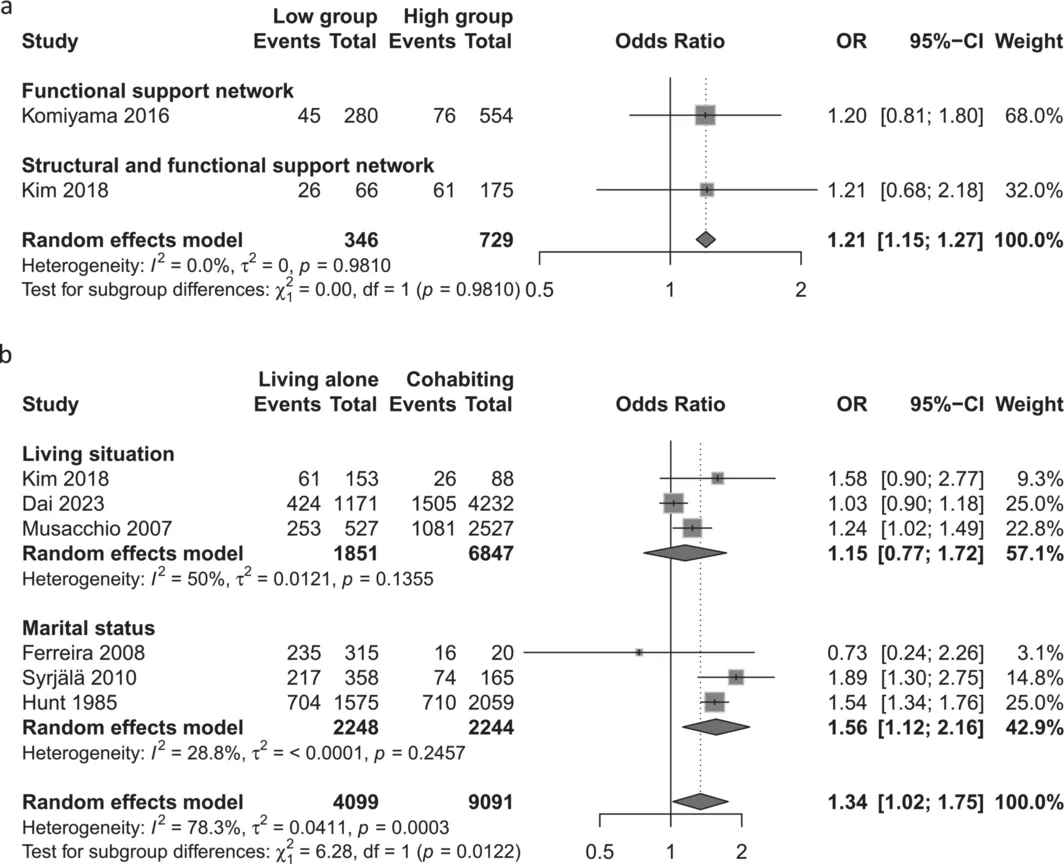
Forest plot of odds ratios for edentulism by levels of (a) functional support and (b) living conditions.

Edentulism was negatively associated with functional support [56, 58] and structural support based on living conditions [43, 45, 51, 56, 61, 67]. Older adults with low levels of functional support were 1.21 (95% CI: 1.15, 1.27; *I* ^2^ = 0%) times and those with low structural support were 1.34 (95% CI: 1.02, 1.75; *I* ^2^ = 78.3%) times more likely to be edentate than their respective counterparts with high levels of support (Figure 3a,b). The studies included in the functional support meta‐analyses were derived from community‐dwelling populations [56, 58], whereas those included in the structural support meta‐analysis were derived from community‐dwelling [51, 56, 61, 67], institutionalised [45] and mixed [43] populations. Population‐stratified analysis of low structural support and edentulism showed an association in community‐dwelling populations (OR 1.48; 95% CI: 1.13, 1.94; *I* ^2^ = 45.6%) [51, 56, 61, 67] (Appendix IV: S3). Sensitivity analysis excluding the most outlier study [43] reduced heterogeneity from *I* ^2^ = 78.3% to 42.4% in the latter meta‐analysis without affecting the pooled estimate (Appendix IV: S4).

**FIGURE 4:**
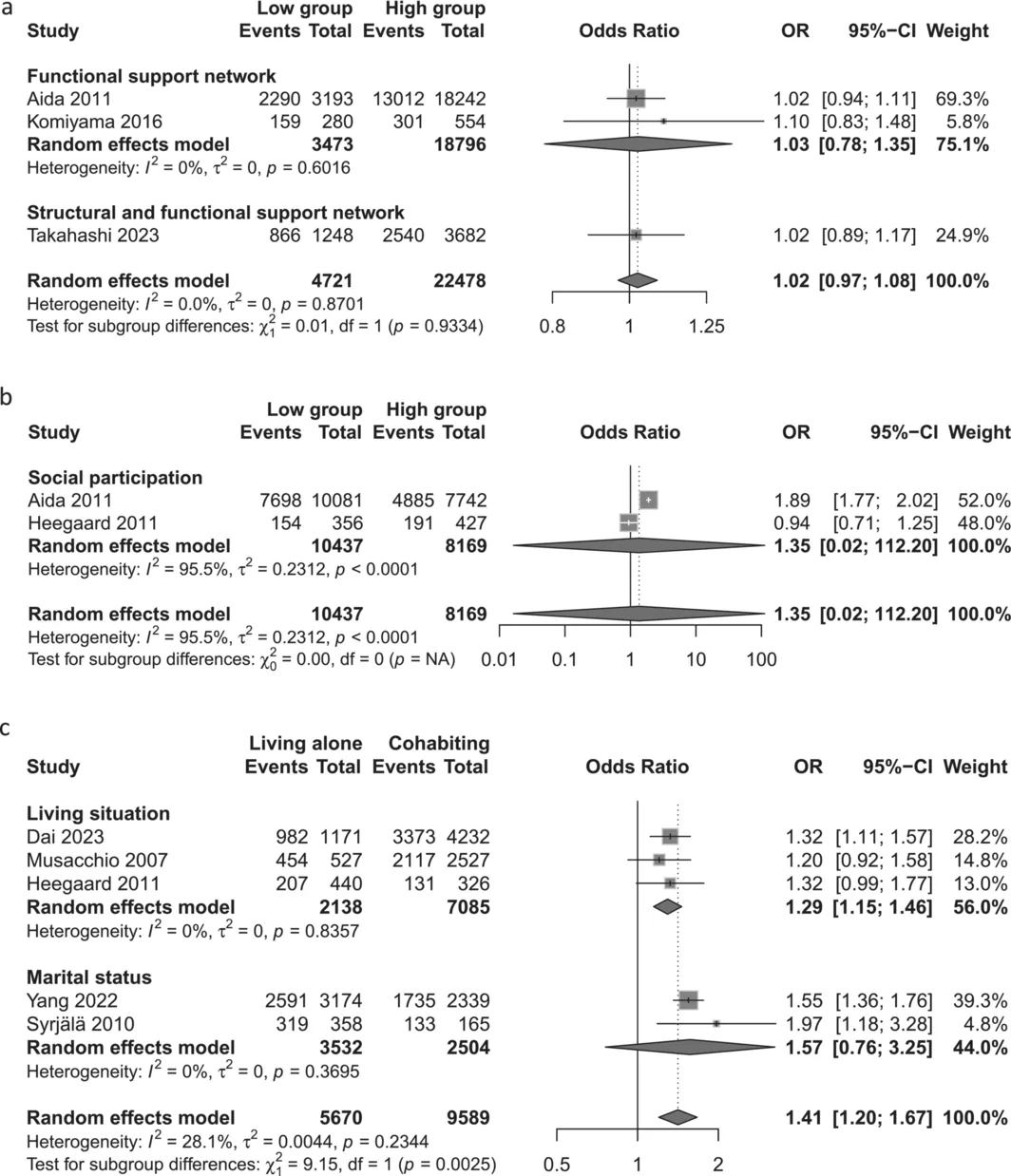
Forest plot of odds ratios for lacking functional dentition (fewer than 20 teeth) by levels of (a) functional support, (b) social participation and (c) living conditions.

Meta‐analyses of functional support [38, 58, 68] and structural support assessed through social participation [38, 48], all derived from community‐dwelling populations, showed no significant associations with functional dentition (Figure 4a,b). In contrast, living alone or being unmarried was associated with higher odds of lacking functional dentition, compared with being married or cohabiting (OR 1.41, 95% CI: 1.20, 1.67; *I* ^2^ = 28.1%) [43, 48, 61, 67, 75] (Figure 4c). This finding was based on studies conducted in community‐dwelling [48, 61, 67] and mixed [43, 75] populations, and the association became evident when these populations were combined (Appendix IV: S5).

In unpooled adjusted analyses, lower levels of functional and structural support were consistently associated with poorer dentition status, including fewer remaining teeth and a higher prevalence of edentulousness [38, 42, 47, 64, 70]. In terms of perceived social support, lower neighbourhood deprivation [46] was associated with better dentition status. Two studies that examined the association of dentition status and loneliness had similar estimates, although the association was significant in one study [68] and non‐significant in the other [64].

#### 3.3.2 Oral Conditions: Dental Caries, Periodontal Disease, Dry Mouth

Due to the wide variation in reported measures within this domain, no meta‐analyses could be conducted. Of the nine studies included [39, 44, 46, 49, 54, 60, 65, 69, 72], four reported adjusted analyses [39, 46, 49, 60].

For dental caries, evidence from one cohort study indicated that low structural support—measured by social participation and living conditions—was associated with a higher odds of dental caries [39]. Other studies found no significant associations in unadjusted analyses of dental caries across different living conditions [44, 72].

Five studies examined periodontal diseases. Four of them reported poorer periodontal status among older adults with low social participation [69], low perceived support [46], or those who were unmarried [65, 72]. Another study showed worse periodontal status among married participants in the unadjusted analysis, but the association was non‐significant after adjustment [49].

For dry mouth, adjusted analyses showed that perceived community‐level social deprivation was associated with a higher prevalence of xerostomia [46], while loneliness was not associated [60]. Using unadjusted data, a study found low social participation to be associated with a higher prevalence of xerostomia [69], while another study found no association between marital status and xerostomia [54].

#### 3.3.3 Oral Function

Five studies were included [56, 59, 60, 68, 69], three of which reported adjusted analyses [56, 60, 68], and no meta‐analyses could be conducted. The largest study showed that low functional or structural support (represented by social isolation) and low perceived support (assessed by loneliness) were significantly associated with difficulty chewing [68]. Another study from Japan documented that low social participation was associated with poorer masticatory function; however, no associations were found with swallowing difficulties, poor tongue mobility, or articulation problems [69]. The remaining studies reported largely similar but non‐significant results [56, 59, 60].

#### 3.3.4 Oral Health Behaviours

Seven studies assessed associations between social support and oral health behaviours [40, 58, 60, 62, 68, 69, 77]. Meta‐analysis demonstrated that older adults with low functional social support were more likely to have non‐regular dental service use (OR 1.20, 95% CI: 1.17, 1.23) than those with high functional support, with no heterogeneity observed across community‐dwelling [58] and institutionalised [62] populations (Appendix IV: S6).

In unpooled analyses, loneliness was also significantly associated with poor dental attendance [60]. However, another study found no significant associations with living situation or marital status and dental attendance [40], while one study showed that married older adults reported significantly worse dental attendance than those who were unmarried [77]. Infrequent toothbrushing was observed in three studies [62, 68, 69]. Of the two studies that provided adjusted analyses, one found an association with loneliness but not with social isolation [68], while the other found no association between infrequent toothbrushing and functional support [62].

#### 3.3.5 OHRQoL

Three studies contributed to the meta‐analysis [41, 57, 71] that showed a non‐significant difference in OHRQoL between older adults being married or cohabiting and those living alone or being unmarried, with a standardised mean difference (SMD) of 0.00 (95% CI: −0.20, 0.20), and substantial heterogeneity (*I* ^2^ = 77.0%) (Appendix IV: S7). This finding was based on studies conducted in community‐dwelling [41, 71] and mixed [57] populations (Appendix IV: S8). When restricting the analysis to studies comparing adults cohabiting versus those living alone, the former group had better OHRQoL than the latter (SMD 0.15, 95% CI: 0.02, 0.28) [71]. Nonetheless, adjusted analyses of unpooled studies showed no significant associations [57, 71, 73, 76].

## 4 Discussion

This is the first systematic review and meta‐analysis that examined the associations between multiple constructs of social support and a wide range of oral health measures among older adults aged 75 years and over. The meta‐analyses showed that low social support, particularly functional and structural support, and adverse living conditions (living alone or without a partner) were consistently associated with poor dentition status (edentulism or lack of functional dentition). Similarly, low functional support was associated with non‐regular dental service use, although this was pooled from only two studies. In contrast, the evidence for other oral health measures was limited and heterogeneous. Most associations in the meta‐analyses were observed in community‐dwelling populations. This finding may indicate that population context plays a role in shaping the observed associations. At the same time, the limited number of studies conducted among institutionalised older adults may partly explain the lack of detected associations in this group.

The strongest and most consistent associations were observed between adverse living conditions (living alone or unmarried) and edentulism and lack of functional dentition. This may imply that low social support could hinder the ability to maintain the natural dentition in this population group. Structural support measured by social participation showed no association with dentition status in meta‐analyses; however, unpooled analyses indicated positive associations with tooth retention. A systematic review and meta‐analysis among adults aged 60 years and over reported that weaker social relationships were associated with a higher risk of having a low number of remaining teeth or edentulism [24]. Others have shown that the results may depend on the characteristics of social support, with positive and quality social support helping to maintain natural dentition in older adults [38, 78].

Social support was associated with oral health behaviours, particularly dental service utilisation. A meta‐analysis that included only two studies showed that lower functional support was associated with non‐regular dental service use. Unpooled analyses further showed associations between poor perceived social support (loneliness) and poor oral hygiene practices, as well as lower non‐regular dental attendance. These findings are consistent with the literature on other populations showing that poor social support was associated with lower dental care utilisation [12, 26, 79, 80]. Our findings highlight the importance of social support in influencing oral health behaviours, particularly for this population. It can facilitate access to dental services by helping people arrange dental appointments through relatives or carers and by assisting in transport [81, 82]. It can also enable healthy behaviours and self‐care [82, 83].

Evidence on the association of social support with oral conditions and oral function was sparse and therefore could not be subjected to meta‐analysis. The general pattern was also heterogeneous. For example, adjusted analyses from a cohort study showed that lower structural support, assessed through social participation, was associated with root caries but not coronal caries, while living alone was associated with coronal caries [39]. There were also inconsistent associations between social support and periodontal disease, dry mouth, and—to a lesser degree—oral function. Compared to the respective findings for dentition status, oral function extends beyond dentition status and is likely influenced also by other factors, such as denture fit, cognitive function, neuromuscular coordination, and dietary adaptation [84, 85, 86]. These complexities may further contribute to heterogeneous findings and limit the ability to detect associations in the existing literature.

Findings on OHRQoL were limited to a single exposure, that is living conditions (living alone and marital status). While the overall meta‐analysis showed no association with OHRQoL, a subgroup analysis showed that living alone was associated with poorer OHRQoL, potentially highlighting the significance of social isolation and loneliness in this age group [29, 30, 87]. The lack of significant or consistent findings should not be interpreted as evidence of no association in this case; rather, it reflected the limited quantity and variability of the existing studies.

This systematic review and meta‐analysis covered multiple aspects of social support and a range of oral health measures in a group of older adults that were not specifically looked at in the existing literature. Moreover, while the review focused on older adults aged 75 years and over, several included studies were not conducted exclusively in this age group, but were included if relevant estimates were reported, allowing inclusiveness of the synthesis of relevant evidence.

There are also several limitations that should be considered. First, the considerable heterogeneity in how social support and oral health were measured restricted the number of meta‐analyses that could be conducted; in several cases, fewer than five studies could be pooled, and these results should be interpreted with caution when considering generalisability. Second, the meta‐analyses relied on unadjusted estimates, limiting the ability to account for confounding, while studies providing only adjusted estimates could not be pooled due to excessive variation in variable selection and adjustment modelling between the different studies. Consequently, the observed associations in the meta‐analyses may be subject to residual confounding, as social support and oral health are influenced by a complex interplay of socioeconomic factors, frailty, cognitive impairment, health behaviours, and multimorbidity. However, to facilitate a more comprehensive interpretation, findings from studies with adjusted estimates were presented narratively following those from the meta‐analysis. Third, most meta‐analyses of structural social support were primarily based on marital status or living arrangements, which represent only one component of structural support. Therefore, these measures may not fully capture the broader structural dimensions of support. They may only indirectly reflect other aspects, such as social participation, network size, or the frequency of social interactions. Furthermore, the use of heterogeneous self‐reported instruments may have introduced measurement variability, which should be considered when interpreting the results. Finally, as the vast majority of the included studies were cross‐sectional, the findings reflect associations and should not be interpreted as evidence of causality.

These findings have implications for practice and research. The consistent associations observed between low structural and functional support and poor dentition status among adults aged 75 years and over suggest that social support may influence long‐term tooth retention through behavioural and access‐related factors. Limited social support may hinder the ability to maintain oral hygiene routines, seek timely dental care, or adapt to functional decline.

From a policy standpoint, community interventions on enhancing social support among older adults should also be considered from an oral health perspective. At the clinical level, social support—or the absence of it—should be assessed when developing person‐centred treatment plans for older adults. Such assessments may help identify individuals at higher risk of poor oral health or irregular dental attendance, allowing for the development of tailored preventive strategies, appropriate recall protocols, or engagement with caregivers and other health professionals. Future research should explore the complex pathways linking social support and oral health, particularly through longitudinal studies, and should also focus on the more vulnerable population of institutionalised older adults.

## 5 Conclusion

This systematic review and meta‐analysis showed that poor social support is associated with poor dentition status and oral health behaviours among older adults aged 75 years and over. Evidence for oral conditions, oral function and OHRQoL was more limited and heterogeneous. These findings underscore the importance of incorporating psychosocial factors into oral health assessment and care planning for this population.

## Author Contributions

F.M., G.T., B.J. and M.S. conceived the initial study idea. S.P. and S.S.S. conducted the research study together and were supported by F.M., G.T., B.J., and M.S. S.P. and S.S.S. prepared the first draft, and all authors were involved in reading, critically revising, editing and approving the final manuscript.

## Funding

The authors have nothing to report.

## Ethics Statement

Ethical approval was not required for this study, as it is a systematic review and meta‐analysis of previously published research data and does not involve the collection of primary data from human participants.

## Consent

The authors have nothing to report.

## Conflicts of Interest

The authors declare no conflicts of interest.

## Supporting information


**Appendix S1:** Detailed search strategy.

**Appendix S2:** List of excluded studies and reasons for exclusion.

**Appendix S3:** Risk of bias and quality assessment of included studies.

**Appendix S4:** Supplementary funnel and forest plots for meta‐analysis.

## Data Availability

The data analysed in this study are derived from previously published studies included in the systematic review. All data supporting the findings of this study are available within the article and its Supporting Information. No new primary data were generated for this study.
