# Supplementary material for: Association of Social Support With Oral Health in Older Adults: A Systematic Review and Meta‐Analysis
Source: Gerodontology. 2026 May 15;43(3):475–94. doi: 10.1111/ger.70072 (PMC13498927; doi:10.1111/ger.70072)
Supplement: Supplementary file 1 [file GER-43-475-s002.docx]

**Appendix I: Search terms**

**Medline (PubMed)**

**P = POPULATION SEARCH TERMS**

("Elder*"[Title/Abstract] OR "Geriatric Population*"[Title/Abstract] OR "Geriatric Patient*"[Title/Abstract] OR “Aging Population"[Title/Abstract] OR “Aged Population"[Title/Abstract] OR "Aged 60"[Title/Abstract] OR "60 year*"[Title/Abstract] OR "Gerodont*"[Title/Abstract] OR "Aged 75"[Title/Abstract] OR "75 year*"[Title/Abstract] OR "Aged 80"[Title/Abstract] OR "80 year*"[Title/Abstract] OR “Senior*"[Title/Abstract] OR “Care-depend*"[Title/Abstract] OR “Dental Care for Aged”[MeSH Terms] OR "Older Adults"[Title/Abstract] OR “Older People” [Title/Abstract])

**AND**

**E/I - SEARCH TERMS for EXPOSURE/INTERVENTION**

(“Social support"[MeSH Terms] OR “Social determinants of health"[MeSH Terms] OR “Socioeconomic factors"[MeSH Terms] OR “Health inequities"[MeSH Terms] OR “Health services accessibility"[MeSH Terms] OR "Health inequity"[Title/Abstract] OR "Oral health disparities"[Title/Abstract] OR "Social isolation"[Title/Abstract] OR "Community engagement"[Title/Abstract] OR "Caregiver support"[Title/Abstract] OR "Family support"[Title/Abstract] OR "Income level"[Title/Abstract] OR "Low-income"[Title/Abstract] OR "High-income"[Title/Abstract] OR "Economic status"[Title/Abstract] OR "Education level"[Title/Abstract]  OR "Health literacy"[Title/Abstract] OR "Access to dental care"[Title/Abstract])

**AND**

**O = OUTCOME SEARCH TERMS**

("Oral Health"[MeSH Terms] OR “Tooth Loss”[MeSH Terms] OR "tooth loss"[Title/Abstract] OR "edent*"[Title/Abstract] OR "missing teeth"[Title/Abstract] OR “Jaw, Edentulous”[MeSH Terms] OR “Tooth demineralization"[MeSH Terms] OR "Caries"[Title/Abstract] OR "Carious"[Title/Abstract] OR "Decay*"[Title/Abstract] OR "DMF-T"[Title/Abstract] OR "DMFT"[Title/Abstract] OR “demineralization"[Title/Abstract] OR “Periodontal Diseases”[MeSH Terms] OR "periodont*"[Title/Abstract] OR “Peri-implant*”[Title/Abstract] OR “Peri-mucositis”[Title/Abstract] OR “Dental pulp diseases"[MeSH Terms] OR “Dentin sensitivity"[MeSH Terms] OR “hypersensitivity"[Title/Abstract] OR “Tooth sensitivity”[Title/Abstract] OR "Quality of Life"[MeSH Terms] OR "oral health-related quality of life"[Title/Abstract] OR "OHRQoL"[Title/Abstract] OR "Oral Health Impact Profile"[Title/Abstract] OR "OHIP"[Title/Abstract] OR "Oral Impact on Daily Performances"[Title/Abstract] OR "OIDP"[Title/Abstract] OR "Self-reported oral health”[Title/Abstract] OR "Perceived oral health”[Title/Abstract] OR “Xerostomia"[MeSH Terms] OR “Mucositis"[MeSH Terms] OR “Stomatitis"[MeSH Terms] OR “Candidiasis, Oral"[MeSH Terms] OR “Xerostomia"[Title/Abstract] OR “Mucositis"[Title/Abstract] OR “Stomatitis"[Title/Abstract] OR “Candidiasis”[Title/Abstract] OR “Hyposalivation"[Title/Abstract] OR “Dry mouth"[Title/Abstract])

**Embase**

**P = POPULATION SEARCH TERMS**

'dental procedure'/exp or ‘elder*’:ti,ab,kw or ‘geriatric population*’:ti,ab,kw or ‘geriatric patient*’:ti,ab,kw or ‘aging population’:ti,ab,kw or ‘aged population’:ti,ab,kw or ‘aged 60’:ti,ab,kw or ‘60 year*’:ti,ab,kw or ‘gerodont*’:ti,ab,kw or ‘aged 75’:ti,ab,kw or ‘75 year*’:ti,ab,kw or ‘aged 80’:ti,ab,kw or ‘80 year*’:ti,ab,kw or ‘senior*’:ti,ab,kw or ‘care-depend*’:ti,ab,kw or ‘older adults’:ti,ab,kw or ‘older people’:ti,ab,kw

**AND**

**E/I - SEARCH TERMS for EXPOSURE/INTERVENTION**

‘social support’/exp or ‘social determinants of health’/exp or ‘socioeconomics’/exp or ‘health disparity’/exp or ‘health care access’/exp or ‘health inequity’:ti,ab,kw or ‘oral health disparities’:ti,ab,kw or ‘social isolation’:ti,ab,kw or ‘community engagement’:ti,ab,kw or ‘caregiver support’:ti,ab,kw or ‘family support’:ti,ab,kw or ‘income level’:ti,ab,kw or ‘low-income’:ti,ab,kw or ‘high-income’:ti,ab,kw or ‘economic status’:ti,ab,kw or ‘education level’:ti,ab,kw or ‘health literacy’:ti,ab,kw or ‘access to dental care’:ti,ab,kw

**AND**

**O = OUTCOME SEARCH TERMS**

'dental health'/exp or 'edentulous jaw'/exp or 'tooth demineralization'/exp or 'periodontal disease'/exp or 'tooth pulp disease'/exp or 'dentin hypersensitivity'/exp or 'oral health related quality of life'/exp or 'oral health impact profile'/exp or 'oral health impact profile 14'/exp or 'oral impacts on daily performance'/exp or 'xerostomia'/exp or 'stomatitis'/exp or 'thrush'/exp or ‘tooth loss’:ti,ab,kw or ‘edent*’:ti,ab,kw or ‘missing teeth’:ti,ab,kw or ‘caries’:ti,ab,kw or ‘carious’:ti,ab,kw or ‘decay*’:ti,ab,kw or ‘DMF-T’:ti,ab,kw or ‘DMFT’:ti,ab,kw or ‘demineralization’:ti,ab,kw or ‘periodont*’:ti,ab,kw or ‘peri-implant*’:ti,ab,kw or ‘peri-mucositis’:ti,ab,kw or ‘hypersensitivity’:ti,ab,kw or ‘tooth sensitivity’:ti,ab,kw or ‘oral health-related quality of life’:ti,ab,kw or ‘OHRQoL’:ti,ab,kw or ‘Oral Health Impact Profile’:ti,ab,kw or ‘OHIP’:ti,ab,kw or ‘Oral Impact on Daily Performances’:ti,ab,kw or ‘OIDP’:ti,ab,kw or ‘self-reported oral health’:ti,ab,kw or ‘perceived oral health’:ti,ab,kw or ‘xerostomia’:ti,ab,kw or ‘mucositis’:ti,ab,kw or ‘stomatitis’:ti,ab,kw or ‘candidiasis’:ti,ab,kw or ‘hyposalivation’:ti,ab,kw or ‘dry mouth’:ti,ab,kw

**Cochrane Central Register of Controlled Trials (CENTRAL)**

**P = POPULATION SEARCH TERMS**

#1 [mh “Dental Care for Aged”]

#2 (elder* or (geriatric NEXT population*) or (geriatric NEXT patient*) or (aging NEXT population) or (aged NEXT population) or (aged NEXT 60) or (60 NEXT year*) or gerodont* or (aged NEXT 75) or (75 NEXT year*) or (aged NEXT 80) or (80 NEXT year*) or senior* or (care NEXT depend*) or (older NEXT adults) or (older NEXT people)):ti,ab,kw

**AND**

**E/I - SEARCH TERMS for EXPOSURE/INTERVENTION**

#3 [mh “Social support”] OR [mh “Social determinants of health”] OR [mh “Socioeconomic factors”] OR [mh “Health inequities”] OR [mh “Health services accessibility”]

#4 ((health NEXT inequity) or (oral NEXT health NEXT disparities) or (social NEXT isolation) or (community NEXT engagement) or (caregiver NEXT support) or (family NEXT support) or (income NEXT level) or (low NEXT income) or (high NEXT income) or (economic NEXT status) or (education NEXT level) or (health NEXT literacy) or (access NEXT to NEXT dental NEXT care)):ti,ab,kw

**AND**

**O = OUTCOME SEARCH TERMS**

#5 [mh “Oral Health”] OR [mh “Tooth Loss”] OR [mh “Jaw, Edentulous”] OR [mh “Tooth Demineralization”] OR [mh “Periodontal Diseases”] OR [mh “Dental Pulp Diseases”] OR [mh “Dentin Sensitivity”] OR [mh “Quality of Life”] OR [mh “Xerostomia”] OR [mh “Mucositis”] OR [mh “Stomatitis”] OR [mh “Candidiasis, Oral”]

#6 ((tooth NEXT loss) or edent* or (missing NEXT teeth) or caries or carious or decay* or (DMF NEXT T) or DMFT or demineralization or periodont* or (peri NEXT implant*) or (peri NEXT mucositis) or hypersensitivity or (tooth NEXT sensitivity) or (oral NEXT health NEXT related NEXT quality NEXT of NEXT life) or OHRQoL or (Oral NEXT Health NEXT Impact NEXT Profile) or OHIP or (Oral NEXT Impact NEXT on NEXT Daily NEXT Performances) or OIDP or (self NEXT reported NEXT oral NEXT health) or (perceived NEXT oral NEXT health) or xerostomia or mucositis or stomatitis or candidiasis or hyposalivation or (dry NEXT mouth)):ti,ab,kw
