# Supplementary material for: Association of Social Support With Oral Health in Older Adults: A Systematic Review and Meta‐Analysis
Source: Gerodontology. 2026 May 15;43(3):475–94. doi: 10.1111/ger.70072 (PMC13498927; doi:10.1111/ger.70072)
Supplement: Supplementary file 2 [file GER-43-475-s003.docx]

**Appendix IV**

**Figure S1** Funnel plot of odds ratios for edentulism by living conditions

**Figure S2** Funnel plot of odds ratios for lacking functional dentition by living conditions

**Figure S3** Forest plot of population-stratified odds ratios for edentulism by living conditions

**Figure S4** Forest plot of odds ratios for edentulism by living conditions (sensitivity analysis)

**Figure S5** Forest plot of population-stratified odds ratios for lacking functional dentition by living conditions

**Figure S6** Forest plot of odds ratios for non-regular dental service use by levels of functional social support

**Figure S7** Forest plot of standardized mean differences in OHRQoL by living conditions

**Figure S8** Forest plot of population-stratified standardized mean difference in OHRQoL by living conditions
