# Supplementary material for: Association of Social Support With Oral Health in Older Adults: A Systematic Review and Meta‐Analysis
Source: Gerodontology. 2026 May 15;43(3):475–94. doi: 10.1111/ger.70072 (PMC13498927; doi:10.1111/ger.70072)
Supplement: Supplementary file 3 [file GER-43-475-s004.docx]

**Appendix III**

Cohort

|  | Selection | | | | Comparability | Outcome | | |  |
| --- | --- | --- | --- | --- | --- | --- | --- | --- | --- |
| Author (Year) | Representativeness of the exposed cohort | Selection of the non-exposed cohort | Ascertainment of exposure | Demonstration that outcome of interest was not present at start of study | Comparability of cohorts on the basis of the design or analysis | Assessment of outcome | Was follow-up long enough for outcomes to occur | Adequacy of follow-up of cohorts | Quality |
| Avlund, K., Holm-Pedersen, P., Morse, D.E. et al. (2003) [1] | * | * | * | - | * | * | * | - | Fair |
| Ganbavale, S.G., Papachristou, E., Mathers, J.C. et al. [2] | * | * | * | - | ** | - | * | - | Fair |
| Qi, X., Pei, Y., Wang, K. et al. [3] | * | * | * | * | ** | - | * | - | Fair |

Cross sectional (association)

|  | Study sample selection | | Assessment of exposure and outcome | | Confounding factors | |  |
| --- | --- | --- | --- | --- | --- | --- | --- |
| Author (Year) | Representativeness of the study sample | Sample size | Assessment of the exposure(s) | Assessment of the outcome(s) | Adjustment for confounder(s) | Assessment of confounder(s) | Risk of bias |
|  |  |  |  |  |  |  |  |
| Aida, J., Kuriyama, S., Ohmori-Matsuda, K. et al. (2011) [4] | * | - | * | * | * | * | Moderate |
| Brothwell, D., Jay, M., and Schonwetter, D. (2008) [5] | * | - | ** | * | * | * | Moderate |
| Ganbavale, S.G., Papachristou, E., Mathers, J.C. et al. (2024) [2] | * | - | * | ** | ** | * | Low |
| Hsu, K-J., Lee, H-E., Wu, Y-M. et al. (2013) [6] | - | - | ** | ** | * | * | Moderate |
| Chan, C.C.K., Li, K.Y., Chen, H. et al. (2025) [7] | * | * | ** | * | ** | * | Low |
| Kim, E-K., Jung, Y-S., Kim, K-R. et al. (2018) [8] | * | - | ** | ** | * | * | Low |
| Kim, H-Y, Jang, M-S, Chung, C-P et al. (2009) [9] | * | - | * | ** | * | * | Moderate |
| Lundgren M., Osterberg, T., Emilson, G. and Steen, B. (1995) [10] | * | - | * | * | * | * | Moderate |
| Musacchio, E., Perissinotto, E., Binotto, P. et al. (2007) [11] | * | - | ** | ** | * | * | Low |
| Niesten, D., Witter, D.J., Bronkhorst, E.M. and Creugers N.H.J. (2017) [12] | * | - | ** | * | * | * | Moderate |
| Osterberg, T., Era, P., Gause-Nilsson, I., and Steen, B. (1995) [13] | * | - | * | * | * | * | Moderate |
| Takahashi, S., Naganuma, T., Kurita, N. et al. (2023) [14] | * | - | ** | * | ** | * | Low |
| Thorstensson, H. and Johansson, B. (2010) [15] | * | - | ** | ** | * | * | Low |
| Tsakos, G., Sheiham, A., Iliffe, S. et al. (2009) [16] | * | - | ** | ** | ** | * | Low |
| Wu, L.L., Cheung, K.Y., Lam, P.Y.P., and Gao, X.L. (2017) [17] | * | * | ** | ** | ** | * | Low |
| Yang, H., Han, R. and Wang, Z. (2022) [18] | * | - | ** | * | ** | * | Low |
| Zini, A. and Sgan-Cohen, H.D. (2008) [19] | - | * | * | ** | ** | * | Low |
| Zini, A., Vered, Y. and Sgan-Cohen, H.D. (2011) [20] | - | * | ** | * | * | * | Moderate |

Cross sectional (prevalence only)

|  | Study sample selection | | Assessment of outcome |  |
| --- | --- | --- | --- | --- |
| Author (Year) | Representativeness of the study sample | Sample size | Assessment of the outcome(s) | Risk of bias |
| Aida, J., Kuriyama, S., Ohmori-Matsuda, K. et al. (2011) [4] | * | * | * | Low |
| Brothwell, D., Jay, M., and Schonwetter, D. (2008) [5] | * | - | * | Moderate |
| Castrejon-Perez, R.C., Borges-Yanez, S.A., Irigoyen-Camacho, M.E. and Cruz-Hervert, L.P. (2017) [21] | * | - | ** | Low |
| Dai M., Song, Q., Lin, T. et al. (2023) [22] | * | * | * | Low |
| Edman, K., Ohrn, K., Nordstrom, B. and Holmlund, A. (2016) [23] | * | - | * | Moderate |
| Ferreira, R.C., Magalhaes, C.S. and Moreira, A.N. (2008) [24] | * | * | * | Low |
| Ganbavale, S.G., Papachristou, E., Mathers, J.C. et al. (2024) [2] | * | - | ** | Low |
| Hatta K., Ikebe, K., Gondo, Y. et al. (2018) [25] | * | - | * | Moderate |
| Heegard, K., Avlund, K., Holm-Pedersen et al. (2011) [26] | * | - | * | Moderate |
| Holm-Pedersen, P., Russell, S.L., Avlund, K. et al. (2006) [27] | * | - | * | Moderate |
| Hsu, K-J., Lee, H-E., Wu, Y-M. et al. (2013) [6] | * | - | ** | Low |
| Hunt, R., Beck, J., Lemke, J. et al. (1985) [28] | * | - | * | Moderate |
| Inomata, C., Ikebe, K., Okubo, H. et al. (2017) [29] | * | - | * | Moderate |
| Islas-Granillo, H., Borges-Yanez, A., Medina-Solis, C. et al. (2016) [30] | * | * | * | Low |
| Chan, C.C.K., Li, K.Y., Chen, H. et al. (2025) [7] | * | * | * | Low |
| Islas-Granillo, H., Borges-Yanez, S.A., Medina-Solis, C.E. et al. (2014) [31] | * | - | ** | Low |
| Jensen, P., Saunders, R., Thierer, T. and Friedman, B. (2008) [32] | * | - | ** | Low |
| Kim, E-K., Jung, Y-S., Kim, K-R. et al. (2018) [8] | * | - | ** | Low |
| Kim, H-Y, Jang, M-S, Chung, C-P et al. (2009) [9] | * | - | ** | Low |
| Komiyama, T., Ohi T., Miyoshi, Y. et al. (2016) [33] | * | - | * | Moderate |
| Liang, YH, Chou, C., Chen, YJ et al. (2020) [34] | - | - | ** | Moderate |
| Lundgren M., Osterberg, T., Emilson, G. and Steen, B. (1995) [10] | * | - | * | Moderate |
| Musacchio, E., Perissinotto, E., Binotto, P. et al. (2007) [11] | * | - | * | Moderate |
| Niesten, D., Witter, D.J., Bronkhorst, E.M. and Creugers N.H.J. (2017) [12] | * | * | ** | Low |
| Osterberg, T., Era, P., Gause-Nilsson, I., and Steen, B. (1995) [13] | * | - | * | Moderate |
| Qi, X., Pei, Y., Wang, K. et al. (2023) [3] | * | * | * | Low |
| Rapp, L., Sourdet, S., Vellas, B. and Lacoste-Ferre, M.H. (2017) [35] | * | - | * | Moderate |
| Rocha-Ortiz, J.A., Tepox-Puga, S.M., Borges-Yanez, S.A. et al. (2023) [36] | - | * | * | Low |
| Takeda, M., Matsuda, Y., Abe, T. et al. (2025) [37] | * | - | * | Moderate |
| Syrjala, A-M. H., Ylostalo, P., Hartikainen, S. et al. (2010) [38] | * | - | ** | Low |
| Takahashi, S., Naganuma, T., Kurita, N. et al. (2023) [14] | * | - | * | Moderate |
| Thorstensson, H. and Johansson, B. (2010) [15] | * | - | * | Moderate |
| Tsakos, G., Sheiham, A., Iliffe, S. et al. (2009) [16] | * | * | ** | Low |
| Wright, FAC., Chu, SK-Y, Milledge, KL. et al. (2017) [39] | * | - | ** | Low |
| Wu, L.L., Cheung, K.Y., Lam, P.Y.P., and Gao, X.L. (2017) [17] | * | * | ** | Low |
| Yanagisawa, S., Sugisawa, H., Harada, K. and Sugihara, Y. (2023) [40] | * | - | * | Moderate |
| Yang, H., Han, R. and Wang, Z. (2022) [18] | * | - | ** | Low |
| Zini, A. and Sgan-Cohen, H.D. (2008) [19] | - | * | ** | Low |
| Zini, A., Vered, Y. and Sgan-Cohen, H.D. (2011) [20] | - | - | ** | Moderate |

References

1.	Avlund, K., et al., *Social relations as determinants of oral health among persons over the age of 80 years.* Community dentistry and oral epidemiology, 2003. **31**(6): p. 454-62.

2.	Ganbavale, S., et al., *Neighborhood Deprivation and Changes in Oral Health in Older Age: A Longitudinal Population-Based Study.* Journal of dental research, 2024. **103**(4): p. 434-441.

3.	Qi, X., et al., *Social isolation, loneliness and accelerated tooth loss among Chinese older adults: A longitudinal study.* Community dentistry and oral epidemiology, 2023. **51**(2): p. 201-210.

4.	Aida, J., et al., *The association between neighborhood social capital and self-reported dentate status in elderly Japanese--the Ohsaki Cohort 2006 Study.* Community dentistry and oral epidemiology, 2011. **39**(3): p. 239-49.

5.	Brothwell, D., M. Jay, and D. Schönwetter, *Dental service utilization by independently dwelling older adults in Manitoba, Canada.* Journal (Canadian Dental Association), 2008. **74**(2): p. 161-161f.

6.	Hsu, K., et al., *Masticatory factors as predictors of oral health-related quality of life among elderly people in Kaohsiung City, Taiwan.* Quality of life research : an international journal of quality of life aspects of treatment, care and rehabilitation, 2014. **23**(4): p. 1395-405.

7.	Chan, C.C.K., et al., *Role of Social Capital on Edentulism, Social Participation and Cognition.* Journal of Dentistry, 2025: p. 106163.

8.	Kim, E.K., et al., *Social capital and oral health: The association of social capital with edentulism and chewing ability in the rural elderly.* Archives of Gerontology and Geriatrics, 2018. **74**: p. 100-105.

9.	Kim, H., et al., *Chewing function impacts oral health-related quality of life among institutionalized and community-dwelling Korean elders.* Community dentistry and oral epidemiology, 2009. **37**(5): p. 468-76.

10.	Lundgren, M., et al., *Oral complaints and utilization of dental services in relation to general health factors in a 88-year-old Swedish population.* Gerodontology, 1995. **12**(12): p. 81-8.

11.	Musacchio, E., et al., *Tooth loss in the elderly and its association with nutritional status, socio-economic and lifestyle factors.* Acta odontologica Scandinavica, 2007. **65**(2): p. 78-86.

12.	Niesten, D., et al., *Oral health care behavior and frailty-related factors in a care-dependent older population.* Journal of dentistry, 2017. **61**: p. 39-47.

13.	Osterberg, T., et al., *Dental state and functional capacity in 75-year-olds in three Nordic localities.* Journal of oral rehabilitation, 1995. **22**(8): p. 653-60.

14.	Takahashi, S., et al., *Social Isolation/Loneliness and Tooth Loss in Community-Dwelling Older Adults: The Sukagawa Study.* Innovation in aging, 2023. **7**(6): p. igad065.

15.	Thorstensson, H. and B. Johansson, *Why do some people lose teeth across their lifespan whereas others retain a functional dentition into very old age?* Gerodontology, 2010. **27**(1): p. 19-25.

16.	Tsakos, G., et al., *The impact of educational level on oral health-related quality of life in older people in London.* European journal of oral sciences, 2009. **117**(3): p. 286‐292.

17.	Wu, L.L., et al., *Oral health indicators for risk of malnutrition in elders.* Journal of Nutrition, Health and Aging, 2018. **22**(2): p. 254-261.

18.	Yang, H., R. Han, and Z. Wang, *Socioeconomics, health-related factors, and tooth loss among the population aged over 80 years in China.* BMC public health, 2022. **22**(1): p. 444.

19.	Zini, A. and H. Sgan-Cohen, *The effect of oral health on quality of life in an underprivileged homebound and non-homebound elderly population in Jerusalem*. Vol. 56. 2008, United States. 99-104.

20.	Zini, A., Y. Vered, and H. Sgan-Cohen, *The association between demographic and oral health-related quality of life factors and dental care attendance among underprivileged older people.* Australasian journal on ageing, 2011. **30**(2): p. 70-6.

21.	Castrejón-Pérez, R.C., et al., *Negative impact of oral health conditions on oral health related quality of life of community dwelling elders in Mexico city, a population based study.* Geriatrics and Gerontology International, 2017. **17**(5): p. 744-752.

22.	Dai, M., et al., *Tooth loss, denture use, and all-cause and cause-specific mortality in older adults: a community cohort study.* Frontiers in public health, 2023. **11**: p. 1194054.

23.	Edman, K., et al., *Prevalence of dental caries and influencing factors, time trends over a 30-year period in an adult population. Epidemiological studies between 1983 and 2013 in the county of Dalarna, Sweden.* Acta odontologica Scandinavica, 2016. **74**(5): p. 385-392.

24.	Ferreira, R., C.d. Magalhães, and A. Moreira, *Tooth loss, denture wearing and associated factors among an elderly institutionalised Brazilian population.* Gerodontology, 2008. **25**(3): p. 168-78.

25.	Hatta, K., et al., *Influence of lack of posterior occlusal support on cognitive decline among 80-year-old Japanese people in a 3-year prospective study.* Geriatrics and Gerontology International, 2018. **18**(10): p. 1439-1446.

26.	Heegaard, K., et al., *Amount and type of alcohol consumption and missing teeth among community-dwelling older adults: findings from the Copenhagen Oral Health Senior study*. Vol. 71. 2011, United States. 318-26.

27.	Holm-Pedersen, P., et al., *Periodontal disease in the oldest-old living in Kungsholmen, Sweden: findings from the KEOHS project.* Journal of clinical periodontology, 2006. **33**(6): p. 376-84.

28.	Hunt, R., et al., *Edentulism and oral health problems among elderly rural Iowans: the Iowa 65+ rural health study.* American journal of public health, 1985. **75**(10): p. 1177-81.

29.	Inomata, C., et al., *Dietary Intake Is Associated with Occlusal Force Rather Than Number of Teeth in 80-y-Old Japanese.* JDR clinical and translational research, 2017. **2**(2): p. 187-197.

30.	Islas-Granillo, H., et al., *Tooth-Loss Experience and Associated Variables among Adult Mexicans 60 Years and Older.* Puerto Rico health sciences journal, 2016. **35**(2): p. 88-92.

31.	Islas-Granillo, H., et al., *Salivary parameters (Salivary Flow, pH and Buffering Capacity) in stimulated Saliva of Mexican Elders 60 Years old and older.* West Indian Medical Journal, 2014. **63**(7): p. 758-765.

32.	Jensen, P., et al., *Factors associated with oral health-related quality of life in community-dwelling elderly persons with disabilities*. Vol. 56. 2008, United States. 711-7.

33.	Komiyama, T., et al., *Association Between Tooth Loss, Receipt of Dental Care, and Functional Disability in an Elderly Japanese Population: The Tsurugaya Project.* Journal of the American Geriatrics Society, 2016. **64**(12): p. 2495-2502.

34.	Liang, Y., et al., *Impact of periodontal disease and chewing ability on the quality of life of the elderly in an affluent community.* Journal of the Formosan Medical Association = Taiwan yi zhi, 2020. **119**(11): p. 1693-1701.

35.	Rapp, L., et al., *Oral Health and the Frail Elderly.* The Journal of frailty & aging.. 2017. **6**(3): p. 154-160.

36.	Rocha-Ortiz, J.A., et al., *Tooth Loss and Associated Factors in Mexican Older Adults in Nursing Homes: A Multicenter Cross-Sectional Study.* BioMed Research International, 2023. **2023**.

37.	Takeda, M., et al. *Relationship Between Oral Function and Social Participation Among Community-Dwelling Older Adults: An Observational Cross-Sectional Study*. in *Healthcare*. 2025. MDPI.

38.	Syrjälä, A., et al., *Number of teeth and selected cardiovascular risk factors among elderly people*. Vol. 27. 2010, England. 189-92.

39.	Wright, F., et al., *Oral health of community-dwelling older Australian men: the Concord Health and Ageing in Men Project (CHAMP).* Australian dental journal, 2018. **63**(1): p. 55-65.

40.	Yanagisawa, S., et al., *[Psychosocial mediators between socioeconomic status and oral health among urban-dwelling older adults]*. Vol. 70. 2023, Japan. 433-441.
